# Supplementary material for: Rapid turnover of life-cycle-related genes in the brown algae
Source: Genome Biol. 2019 Feb 14;20:35. doi: 10.1186/s13059-019-1630-6 (PMC6374913; doi:10.1186/s13059-019-1630-6)
Supplement: Supplementary file 3 — Protein alignments, corresponding Gblocks html file and CDS sequences for the lineage conserved gametophyte-biased genes. (ZIP 2109 kb) [file 13059_2019_1630_MOESM3_ESM.zip › Con_GA_alignments/Ec-01_000650.aa_ali.fasta-gb.htm]

Ec-01\_000650.aa.fasta.trans.aa\_ali.fasta


## Gblocks 0.91b Results

Processed file: **Ec-01\_000650.aa.fasta.trans.aa\_ali.fasta**  
Number of sequences: **2**  
Alignment assumed to be: **Protein**  
New number of positions: **525** (selected positions are underlined in blue)

```
                         10        20        30        40        50        60
                 =========+=========+=========+=========+=========+=========+
Ec-01_000650     MGLCFIIATVCLTPLVAAITSSSARTDVDLQDLASSISAAREEARVDGRTQPQQQQGLSL
MSTRG.18342.1    --------------XFRAILDSQWR-LLALQDYG-------IESYVS-------------
                                                                             


                         70        80        90       100       110       120
                 =========+=========+=========+=========+=========+=========+
Ec-01_000650     QHLEAMWHTSLGVQLDDSSSVREEAYRSIVDLQWALLASQDDATQTQQSSSLAGQTSESD
MSTRG.18342.1    -----------------ASSVSSKGG--------------DDGNLPDTT-----KEGASD
                                                                           ##


                        130       140       150       160       170       180
                 =========+=========+=========+=========+=========+=========+
Ec-01_000650     SLTPLSRSPFLVCGDRHPDAVLAVESVVHRARTQLAYSRGDTVCLLAGLRLDEVDTVRRS
MSTRG.18342.1    KSRPPLRSPFLVCGNRHPDAILALENVVHRTRAQLTYSRGDTACLLVGLRLDEVEAVRQS
                 ############################################################


                        190       200       210       220       230       240
                 =========+=========+=========+=========+=========+=========+
Ec-01_000650     ETVHVVDPLPQPAKLSRSLHAKLD---EPPKSPVGLVNNDSFFPSNRNSGDGETRKRVLT
MSTRG.18342.1    GGIHVVEPLPQPAKLSRSLHAKLDGHLEPPESST--VRDDASSPPSNASGDEARNTRVAP
                 ########################            ######################  


                        250       260       270       280       290       300
                 =========+=========+=========+=========+=========+=========+
Ec-01_000650     -AAATERAGGVPSPAAQQTGSGETMISDNDSQQGQQ-----LRRVRFNHGEGLPSDLDVS
MSTRG.18342.1    GDTAGEFAGTLRKPRLDPKKQYTFRHSGDDEQQQQQQRVSTPRNVEFNHGEGLPSDLDVS
                    ###########            ##########      ##################


                        310       320       330       340       350       360
                 =========+=========+=========+=========+=========+=========+
Ec-01_000650     LTPGTWGLDMAHTWIEHLTSFESTAHFWCEHLRDRFVWTRHSPVDED----EGAVREGAA
MSTRG.18342.1    LTPGTRDRDIANTWMRHLTSFESTAHLWDEHLRERFLWTRRSAVKEVGVVEESPVGGGNT
                 ##############################################     #########


                        370       380       390       400       410       420
                 =========+=========+=========+=========+=========+=========+
Ec-01_000650     DSDGNERRGLNVDEAPTSQTRMHRTWNTLIESHGLTDLGSLWEQTVDQSSKDGACDFRRL
MSTRG.18342.1    ESEGGRARRSLREEALTSKARLYRDRNALVEAHGLEDVESLWDQTTELSSEDGACDFRRL
                 ############################################################


                        430       440       450       460       470       480
                 =========+=========+=========+=========+=========+=========+
Ec-01_000650     GASSAPEESSKAGSNTKDSKEGAGPHGTTRQDRRPSPSHKGPVG-KKRR--EGGANDRVV
MSTRG.18342.1    HPSSTSELNAP-ASDLRNSRDEATVPRRTRQRREGGSAERPPKGSATHRESGGGTHERIV
                 #######      ###############################        ########


                        490       500       510       520       530       540
                 =========+=========+=========+=========+=========+=========+
Ec-01_000650     LRGADSLGNTPEDNAHCLLTVLAYLVTRPEVSYVDDLPQVFELNVEAAWITQSGEETAYS
MSTRG.18342.1    LRGVGSLGATPQDNPHCLLTLLAYLATLPEVAYVDDLPQVFELNVEAAWITQSGEETSYS
                 ############################################################


                        550       560       570       580       590       600
                 =========+=========+=========+=========+=========+=========+
Ec-01_000650     MWNEGIDGSTEIIAMADSGLDVNSCFFTEDLTDDNIDCSSWSNPVFDLTKRKIVQYVGFV
MSTRG.18342.1    IWDQGIDGRTEIITMADSGLDINSCFFSEDDTDDNIECSTYSSPVYDLTKRKVVQYVGYV
                 ############################################################


                        610       620       630       640       650       660
                 =========+=========+=========+=========+=========+=========+
Ec-01_000650     DCVDTENGHGTHVAGSAAGGLSNSSPGEHFGDGAGFGAKLAVFDFGNSEGGLVTPSSLKS
MSTRG.18342.1    DCGDTENGHGTHVGGSAVGAISNTSPGEHFGDGSGFGAKLAVFDFGDEDGSLVTPSNIQT
                 ############################################################


                        670       680       690       700       710       720
                 =========+=========+=========+=========+=========+=========+
Ec-01_000650     QMLQPPYDIGARISTNSWGAVRTTYTSLDRQVDDFAYLNQDMVIVIAAGNCGDEISGCQY
MSTRG.18342.1    QMLQPAYDIGARISTNSWGAIRTTYTSLDRQMDDFAYFKX--------------------
                 #####################################                       


                        730       740       750       760       770       780
                 =========+=========+=========+=========+=========+=========+
Ec-01_000650     QNADIYGSGSILSPALGKNIISVGASEASPSTAGLADNVDEVAYFSSQGPTVDDNRIKPD
MSTRG.18342.1    ------------------------------------------------------------
                                                                             


                        790       800       810       820       830       840
                 =========+=========+=========+=========+=========+=========+
Ec-01_000650     VVAPGYHLFSASAKPDQESSCEIGPMAGTSMATPVVAGAAAMIRQYFVQGWYPSGIATPS
MSTRG.18342.1    ------------------------------------------------------------
                                                                             


                        850       860       870       880       890       900
                 =========+=========+=========+=========+=========+=========+
Ec-01_000650     NIFNPSNALVKAMIVTSGTAMASYDSSAGSTTLGSPPDAIQGFGRVLLDSVLNVRGSVDL
MSTRG.18342.1    ------------------------------------------------------------
                                                                             


                        910       920       930       940       950       960
                 =========+=========+=========+=========+=========+=========+
Ec-01_000650     FVEDRVDMTHGDVNSHFIEMDGVSSSAFDGDMKVTMVWMEPAGFSGSVAPVFNDLDLKVV
MSTRG.18342.1    ------------------------------------------------------------
                                                                             


                        970       980       990      1000      1010      1020
                 =========+=========+=========+=========+=========+=========+
Ec-01_000650     AYDGTSESTFYPNNLAAKDPDNTVEMVVIPDVDSYEWFNVTVTAASVTTTSLPYYALVVT
MSTRG.18342.1    ------------------------------------------------------------
                                                                             


                       1030      1040      1050      1060      1070      1080
                 =========+=========+=========+=========+=========+=========+
Ec-01_000650     GGVAGTYSPTASTARSWFVRWEMDIDGIPVAQVATEAFEAGLAEAISAETGDYTDETSSV
MSTRG.18342.1    ------------------------------------------------------------
                                                                             


                       1090      1100      1110      1120      1130      1140
                 =========+=========+=========+=========+=========+=========+
Ec-01_000650     VVKIDHSTASASDDRSTVTVEIVVASEADGYAVLTYIISFVEGTSSSVAEGNQGLVAALN
MSTRG.18342.1    ------------------------------------------------------------
                                                                             


                       1150      1160      1170      1180      1190      1200
                 =========+=========+=========+=========+=========+=========+
Ec-01_000650     EALESRTTEGCACVEVQAIDGGGYDCSACEEGAVFSTASSITTMSPGGYDLLSVDRSSGR
MSTRG.18342.1    ------------------------------------------------------------
                                                                             


                       1210      1220      1230      1240      1250      1260
                 =========+=========+=========+=========+=========+=========+
Ec-01_000650     YPVSVFASASDNSDADDDGMFWSDLSGGKTIRGIIRSDSYVHMAGVVAAIVLFTSLGLFA
MSTRG.18342.1    ------------------------------------------------------------
                                                                             


                       1270      1280      1290      1300      1310      1320
                 =========+=========+=========+=========+=========+=========+
Ec-01_000650     LRRGRLDAEGRRLSSTSGSGGDGGVSPQRGKARLSDTGTTQSVADKKASGSKRRDDSREI
MSTRG.18342.1    ------------------------------------------------------------
                                                                             


                 
                 =========
Ec-01_000650     FTPQATPAP
MSTRG.18342.1    ---------
```

```
Parameters used
Minimum Number Of Sequences For A Conserved Position: 2
Minimum Number Of Sequences For A Flanking Position: 2
Maximum Number Of Contiguous Nonconserved Positions: 8
Minimum Length Of A Block: 10
Allowed Gap Positions: None
Use Similarity Matrices: Yes
```

```
Flank positions of the 8 selected block(s)
Flanks: [119  204]  [217  238]  [244  254]  [267  276]  [283  346]  [352  427]  [434  464]  [473  697]  

New number of positions in Ec-01_000650.aa.fasta.trans.aa_ali.fasta-gb:  525  (39% of the original 1329 positions)
```
